# Genetic Knockdown and Pharmacological Inhibition of Parasite Multidrug Resistance Transporters Disrupts Egg Production in *Schistosoma mansoni*

**DOI:** 10.1371/journal.pntd.0001425

**Published:** 2011-12-06

**Authors:** Ravi S. Kasinathan, William M. Morgan, Robert M. Greenberg

**Affiliations:** Department of Pathobiology, School of Veterinary Medicine, University of Pennsylvania, Philadelphia, Pennsylvania, United States of America; University of Queensland, Australia

## Abstract

P-glycoprotein (Pgp) and multidrug resistance-associated proteins (MRPs) are ATP-dependent transporters involved in efflux of toxins and xenobiotics from cells. When overexpressed, these transporters can mediate multidrug resistance (MDR) in mammalian cells, and changes in Pgp expression and sequence are associated with drug resistance in helminths. In addition to the role they play in drug efflux, MDR transporters are essential components of normal cellular physiology, and targeting them may prove a useful strategy for development of new therapeutics or of compounds that enhance the efficacy of current anthelmintics. We previously showed that expression of *Schistosoma mansoni* MDR transporters increases in response to praziquantel (PZQ), the current drug of choice against schistosomiasis, and that reduced PZQ sensitivity correlates with higher levels of these parasite transporters. We have also shown that PZQ inhibits transport by SMDR2, a Pgp orthologue from *S. mansoni*, and that PZQ is a likely substrate of SMDR2. Here, we examine the physiological roles of SMDR2 and SmMRP1 (the *S. mansoni* orthologue of MRP1) in *S. mansoni* adults, using RNAi to knock down expression, and pharmacological agents to inhibit transporter function. We find that both types of treatments disrupt parasite egg deposition by worms in culture. Furthermore, administration of different MDR inhibitors to *S. mansoni*-infected mice results in a reduction in egg burden in host liver. These schistosome MDR transporters therefore appear to play essential roles in parasite egg production, and can be targeted genetically and pharmacologically. Since eggs are responsible for the major pathophysiological consequences of schistosomiasis, and since they are also the agents for transmission of the disease, these results suggest a potential strategy for reducing disease pathology and spread.

## Author Summary

Schistosomes are parasitic flatworms that are the causative agents of schistosomiasis, a major tropical disease. As adults, schistosomes reside within the host vasculature, taking up nutrients, evading host defenses, and expelling wastes and toxins. Multidrug resistance transporters are involved in removal of toxins and foreign compounds, including drugs, from cells. These transporters have broad selectivity, and when upregulated or mutated, can confer resistance to a wide spectrum of drugs against mammalian tumor cells. They are also associated with drug resistance in various parasites, including helminths. In this report, we have used knockdown of expression of these proteins and pharmacological inhibition of their transport function to dissect their physiological role in the schistosome life cycle. We find that either reducing transporter expression or pharmacologically inhibiting transporter function leads to disruption of egg production by adult worms. Eggs deposited within the host are the major cause of disease pathology, and eggs excreted by the host are the means of continuation of the life cycle and transmission of the disease. The capability to interfere with schistosome egg production could have major implications for development of new treatment strategies.

## Introduction

Schistosomiasis is a major endemic disease that affects hundreds of millions worldwide, causes nearly 300,000 deaths annually, and has an estimated human health burden on a par with malaria or tuberculosis [1]–[3]. The causative agents of schistosomiasis are parasitic flatworms of the genus *Schistosoma*. Adult schistosomes reside in the vasculature of the host, where they take up nutrients and deposit eggs which evoke a host immunopathological response that is responsible for the development of the pathophysiological effects of chronic schistosomiasis. Like other organisms, schistosomes must eliminate toxic metabolites and xenobiotics, and, as parasites, must in addition deal with potentially toxic compounds generated by the host [4].

Multidrug resistance (MDR) proteins are cellular efflux transporters with broad substrate specificities that likely play essential roles in this process, as well as in other significant aspects of parasite physiology. Several of these transporters are members of the ATP binding cassette (ABC) superfamily of proteins, including P-glycoprotein (Pgp), multidrug resistance-associated proteins (MRPs), breast cancer resistance protein (BCRP), and others [5], [6]. Their major role in normal cellular physiology is to remove or exclude xenobiotics and metabolic toxins, but they are also involved in a wide array of physiological functions [7]–[9], including regulation of cell death [10] and immune function [11].

As their name suggests, MDR transporters also mediate multidrug resistance, a phenomenon in which cells that develop resistance to a particular drug also show unexpected cross-resistance to several structurally unrelated compounds. Though MDR transporter-mediated multidrug resistance was described originally in mammalian cells [12], MDR transporter expression levels and allele frequencies are also altered in anthelmintic-resistant populations of helminths, including schistosomes [13]–[22]. The role these transporters might be playing in helminth and other parasite drug resistance has recently been reviewed [23]–[27].

Praziquantel (PZQ) is the current drug of choice against schistosomiasis. It is highly effective against all schistosome species, and shows minimal adverse effects [28]–[30]. However, schistosomes show stage- and sex-dependent differences in susceptibility to PZQ [31]–[33], and the mode of action of the drug remains unresolved three decades following its introduction [34], [35]. Though currently there is little compelling evidence that PZQ resistance constitutes a major problem in the field, several reports of worm isolates exhibiting reduced PZQ susceptibility following drug pressure have appeared in the literature, and could be harbingers of the emergence of more widespread resistance [36]–[38]. Recent studies on changes in gene expression in response to PZQ may provide clues to the mode of action of the drug and to possible molecular mechanisms underlying development of resistance [39], [40].

ABC transporter cDNAs that have been characterized in schistosomes include SMDR2 [41], a *S. mansoni* orthologue of Pgp, and SmMRP1 [42], a *S. mansoni* orthologue of MRP1. SMDR2 RNA is expressed at higher levels in female parasites than in males [21], [41], while males express higher SmMRP1 RNA levels than females [42]. Notably, *S. mansoni* adults upregulate expression of both of these transporters in response to PZQ [21], [42]. Furthermore, higher basal levels of both SMDR2 and SmMRP1 correlate with reduced PZQ susceptibility [21], [42], and PZQ inhibits, and is also a likely substrate of, SMDR2 [43]. Based on these findings, we have hypothesized that schistosome MDR transporters may be modulating the responsiveness of parasites to PZQ [44]. We also predict that schistosome multidrug transporters play critical roles in worm physiology, development, and perhaps in modifying host responses.

In this report, we use genetic and pharmacological approaches to examine the effects on schistosomes of interference with normal MDR transporter function. We find that knockdown of SMDR2 or SmMRP1 expression in adult worms, or exposure of parasites to pharmacological inhibitors of these transporters, disrupts egg production in *S. mansoni* cultured *ex vivo*. Furthermore, administration of any of four structurally diverse Pgp inhibitors to schistosome–infected mice results in a reduced egg burden in the livers of those infected mice. Schistosome eggs are associated with the majority of morbidity in chronic schistosomiasis, and are the agents of disease transmission. Our findings indicate that MDR transporters may be essential components of pathways involved in schistosome reproduction, and may serve as highly “drugable” targets for new antischistosomals that decrease egg-dependent pathology and could serve to reduce disease transmission.

## Results

### Knockdown of SMDR2 and SmMRP1 RNA and protein in *S. mansoni* adults

**Figure 1:**
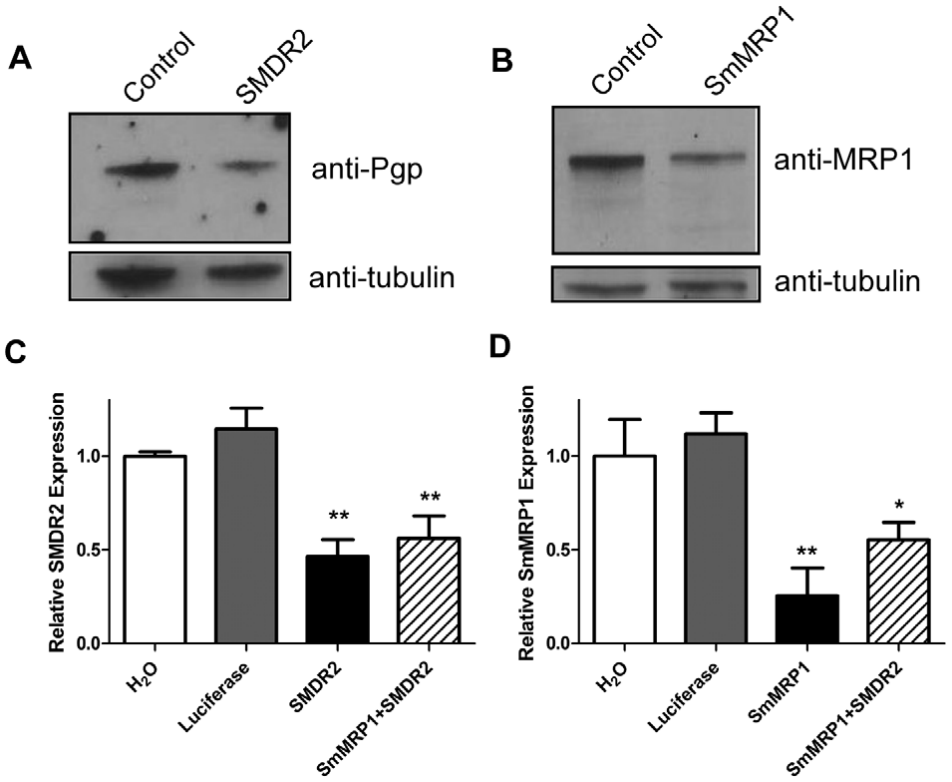
Knockdown of SMDR2 and SmMRP1 expression in adult parasites. Adult parasites were perfused at 6–7 weeks post infection and electroporated with 3 µg of siRNAs or water. Following electroporation, pooled adult worms (males and females) were incubated as described in Materials and Methods, and the expression of SMDR2 and SmMRP1 analyzed for changes in RNA and protein abundance (A, B). Western blot analysis of anti-Pgp (A) or anti-MRP1 (B) cross-reactive proteins (upper panel) isolated from worms treated with SMDR2 siRNA (A, lane 2), SmMRP1 siRNA (B, lane 2), or water (Control, lane 1). Note the decrease in immunoreactivity for both target sequences. Anti-β-tubulin was used as a loading control. (C, D) Relative expression of SMDR2 (n = 6–7) or SmMRP1 (n = 3–4) RNA in adult worms treated with water (H_2_O, white bars), luciferase siRNA (grey bars), SMDR2 siRNA or SmMRP1 siRNA (black bars), or both SMDR2 and SmMRP1 (hatched bars). SMDR2 and SmMRP1 siRNAs efficiently knock down the mRNA expression levels of SMDR2 by ≥50% and SmMRP1 by ≥70%, respectively. The fold changes were determined by quantitative RT-PCR using 18S RNA as the reference gene. *, ** indicate P<0.05 and P<0.01, respectively, compared to the water control, ANOVA.

We used electroporation of SMDR2 and SmMRP1 siRNAs to knock down expression of the multidrug resistance proteins SMDR2 and SmMRP1 in adult worms. As shown in Fig. 1, electroporation of adult parasites with siRNA targeted against either sequence results in substantial reduction of the relative expression level of that gene, both at the RNA and protein levels. Levels of RNA expression for both genes in pooled adult schistosomes are reduced by 50–70% compared to controls. Addition of SmMRP1 siRNA to the SMDR2 siRNA does not appear to affect RNA levels of SMDR2, nor does addition of SMDR2 siRNA appear to additionally decrease levels of SmMRP1 RNA. Protein expression, as measured by immunoblotting with anti-Pgp and anti-MRP1 antibodies, is also reduced.

### Knockdown of SMDR2 or SmMRP1 decreases egg production in *S. mansoni* adults

**Figure 2:**
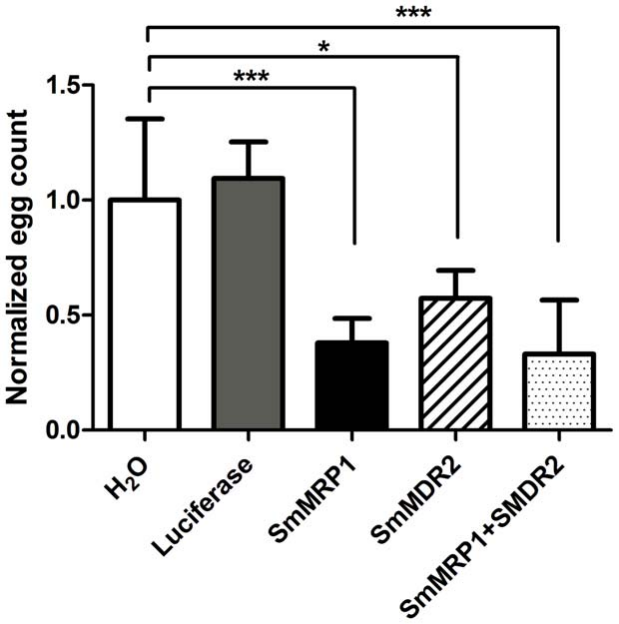
Knockdown of SMDR2 or SmMRP1 in adult schistosomes disrupts parasite egg production. Adult schistosomes were electroporated with H_2_O or 3 µg siRNAs and incubated in RPMI medium for 48 h. Following electroporation, 2–3 adult pairs (n = 4–7) were cultured in 16-well plates for 4–5 days and the number of eggs counted. RNAi treatments were luciferase siRNA (grey bar), SmMRP1 siRNA (black bar), SMDR2 siRNA (hatched bar), or both SmMRP1 and SMDR2 siRNA (dotted bar). Egg counts within each experiment were normalized to the corresponding worms treated with H_2_O (white bar). Treatment with the MDR transporter siRNAs significantly reduced egg production by ≥ 60%, but no significant change in egg production was found for worms electroporated with luciferase siRNA or H_2_O. *, *** indicate P<0.05 and P<0.001, respectively, ANOVA.

Adult schistosomes perfused from the murine host and maintained *in vitro* will continue to produce eggs, though only those deposited during the first 48 h following perfusion from the host appear to be viable [45]. We compared the cumulative number of eggs produced by worms over a 2–3-day span following electroporation with siRNA against SMDR2 or SmMRP1 (or both). We also counted eggs produced by control worms electroporated with luciferase siRNA or with no treatment. As shown in Fig. 2, knockdown of either MDR transporter gene (or both) resulted in a significant reduction in cumulative egg production compared to controls.

### Exposure of adult *S. mansoni* to MDR inhibitors disrupts egg production

As shown above, knockdown of MDR transporter expression in adult *S. mansoni* results in decreased parasite egg production. Previous work described in a patent [46] showed that exposure of worms to verapamil, a mammalian L-type voltage-gated Ca^2+^ (Ca_v_) channel blocker and also an inhibitor of SMDR2 [43] and mammalian Pgp [47], [48], reduces egg production. We have confirmed these results for verapamil, finding no eggs whatsoever following incubation of adults in 10 µM verapamil for 2 days.

**Figure 3:**
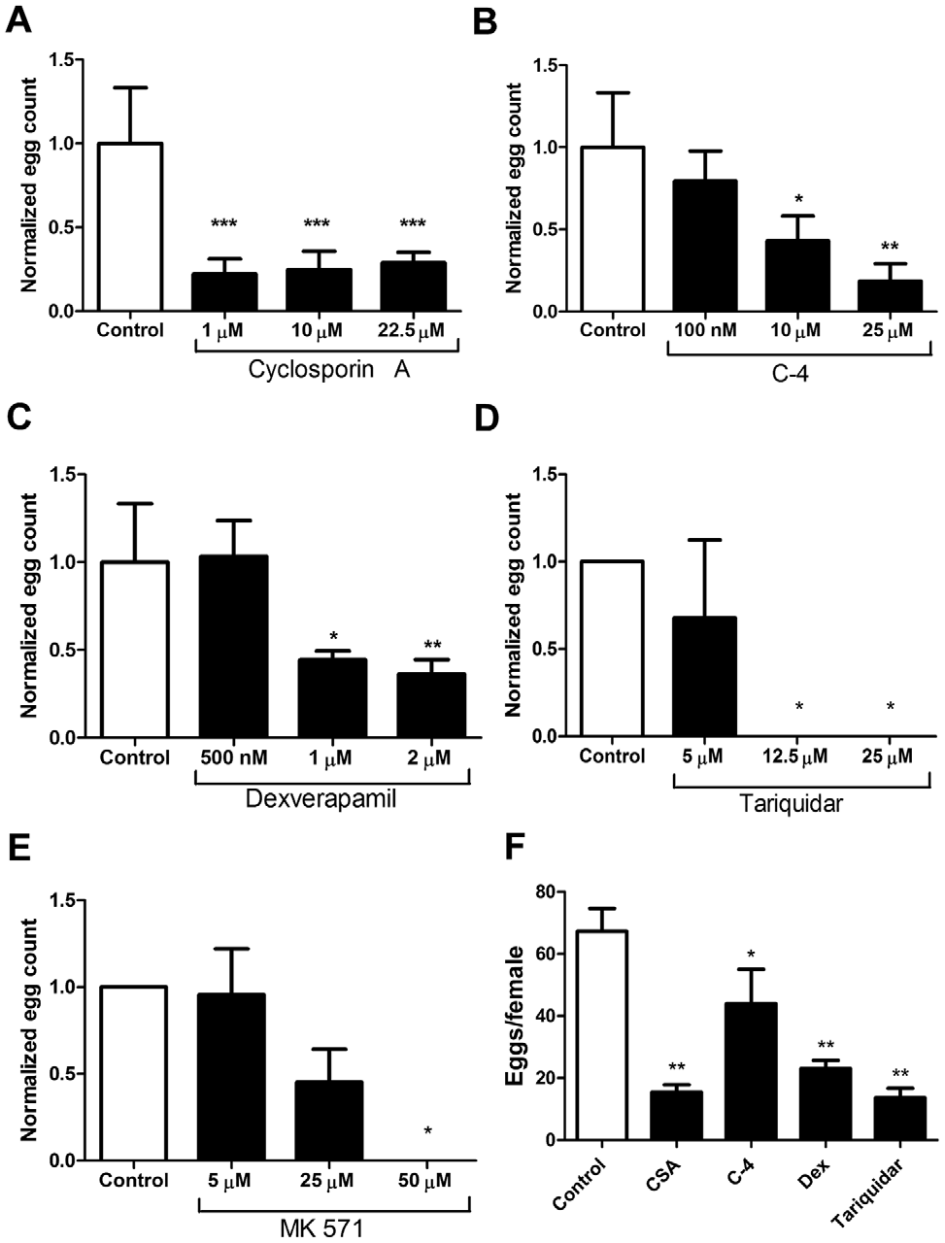
Exposure of *S. mansoni* adult worms in culture to MDR inhibitors disrupts egg production. Adult worm pairs (n = 3–4) were incubated in different concentrations of MDR inhibitors (black bars) for 48 h. Cumulative egg counts were normalized to those of control worms (white bars), which were exposed to DMSO carrier. Addition of the Pgp inhibitors C-4 (A), dexverapamil (B), cyclosporin A (C), tariquidar (D), or the MRP-1 inhibitor MK 571 (E) significantly disrupts egg production. (F) The effect of the Pgp inhibitors on egg production by females is independent of presence of male worms. Adult females were incubated in the absence of males for 48 h in the culture media alone (Control), or in the presence of 10 µM cyclosporin A (CSA), tariquidar, dexverapamil, or C-4. Shown are the cumulative egg counts per female, n = 3–5 for each treatment. *, **, and *** indicate P<0.05, P<0.01, and P<0.001 respectively, ANOVA.

Based on these results, we examined other structurally diverse Pgp and MRP1 inhibitors for their effects on *S. mansoni* egg production. Drugs tested included: the immunosuppressant cyclosporin A (CSA), which is also an inhibitor of mammalian Pgp; R(+)-verapamil (dexverapamil), an enantiomer of verapamil which is significantly less active than the S(−) enantiomer against Ca_v_ channels, but which retains potent and selective competitive inhibitory activity against Pgp [49]; C-4, a curcumin derivative that is a cell-permeable, reversible Pgp inhibitor [50]; tariquidar (aka XR9576), a third-generation, selective and highly potent Pgp inhibitor [51]–[53] (which also appears to be a substrate of BCRP at low concentrations and an inhibitor of BCRP at >100 nM concentrations [54]); and MK 571, a potent inhibitor of MRP1 [55]. As shown in Fig. 3, exposure of adult worms to any of these compounds *ex vivo* resulted in a dramatic, dose-dependent reduction in cumulative parasite egg production over two (tariquidar, MK 571) or five (CSA, dexverapamil, C-4) days in culture. Specifically, exposure of worms to CSA (Fig. 3A) results in a ∼75% decrease in egg production at concentrations of 1 µM–22.5 µM. Worms exposed to C-4 (Fig. 3B) show a 62% decrease at 10 µM and a 92% decrease at 25 µM concentrations, while dexverapamil (Fig. 3C) produces a ∼65% decrease at 1–2 µM. Exposure of parasites to tariquidar at concentrations ≥12.5 µM results in no eggs being deposited whatsoever (Fig. 3D), and in apparent worm lethality (absence of movement or response to stimuli) after 72 h exposure (data not shown). The MRP1 inhibitor MK 571 also disrupts egg production, with no eggs deposited following exposure to 50 µM MK 571 (Fig. 3E). Disruption of egg production also occurs when females cultured alone are exposed to Pgp inhibitors (Fig. 3F). Drug-treated females could not be rescued by addition of untreated males; egg production was still inhibited (data not shown), indicating that the process being targeted is likely autonomous to the female worms or the eggs themselves. Drug treatment, exemplified by dexverapamil, appears to affect the morphology of female reproductive organs (Fig. S1).

**Figure 4:**
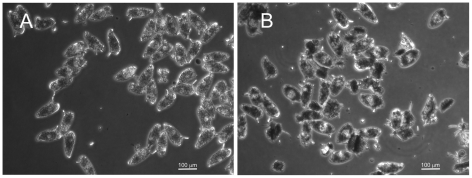
Eggs produced by worms exposed to MDR inhibitors show morphological abnormalities. Micrographs of *S. mansoni* eggs collected from adult worm pairs cultured in the absence (A) or presence (B) of tariquidar for 48 h. Control eggs appear normal and oval shaped with a lateral spine, in contrast to malformed, necrotic, and disintegrated eggs from worms exposed to tariquidar.

Those eggs that were deposited by worms treated with MDR inhibitors such as tariquidar were often morphologically abnormal (Fig. 4), appearing malformed, necrotic, and sometimes disintegrated or in the process of fragmentation. However, other than C-4, the Pgp inhibitors do not appear to be acting on the eggs themselves. Thus, eggs isolated from infected mouse livers and subsequently exposed to CSA, dexverapamil, or tariquidar hatch normally (data not shown). Eggs exposed to C-4 do not appear to hatch.

### Administration of Pgp inhibitors to *S. mansoni*-infected mice decreases liver egg burden

**Figure 5:**
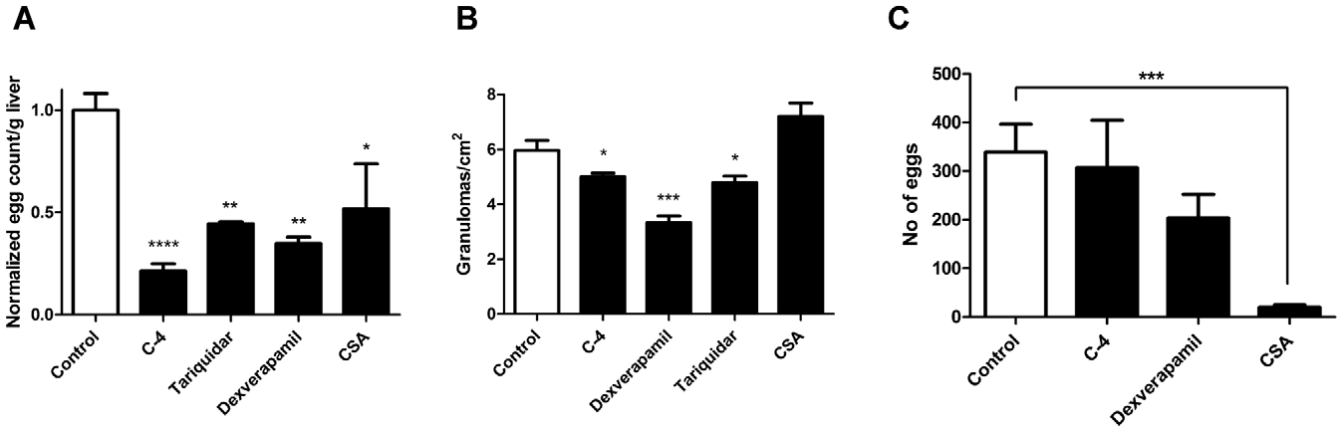
Administration of MDR inhibitors to *S. mansoni*-infected mice reduces host liver egg burden. (A). Mean egg burden/g of liver (n = 3–5) from mice at 6–7 weeks post infection with approximately 200 cercariae, normalized to Control within each experiment. Mice were treated with 3 doses on alternating days of: diluted DMSO/Cremophore EL carrier (Control; white bar, n = 8); C-4 (50 mg/kg, n = 6); tariquidar (15 mg/kg, n = 3); dexverapamil (60 mg/kg, n = 3), or cyclosporin A (CSA, 60 mg/kg, n = 6). (B) Granulomas/cm^2^ found in livers of infected mice (n = 6) treated with carrier or drugs, as in A. (C) Mean *ex vivo* egg production (n = 4–6) from 3 pairs of adult parasites perfused from mice that were treated with the MDR inhibitors dexverapamil (60 mg/kg), C-4 (50 mg/kg), or cyclosporin A (CSA; 60 mg/kg) and subsequently cultured in RPMI for 48 h. Control represents eggs from parasites perfused from mice treated with carrier alone (diluted DMSO/Cremophore EL). Only CSA continues to disrupt egg production through the culture period. *, **, ***, and **** indicate P<0.05, P<0.01, P<0.001, and P<0.0001, respectively, unpaired, two-tailed *t*-tests.

To test whether MDR inhibitors would also disrupt egg production by parasites within the murine host, we administered three intraperitoneal doses (100 µl volume each) of either CSA (60 mg/kg), C-4 (50 mg/kg), dexverapamil (60 mg/kg), or tariquidar (15 mg/kg) to *S. mansoni*-infected mice at 5–6 weeks post-infection. Livers of infected mice treated with any of the four Pgp inhibitors showed significantly reduced egg burden compared to the vehicle-injected control (Fig. 5A). Egg burden was reduced approximately 80% following administration of C-4, 65% following administration of dexverapamil, 55% following administration of tariquidar, and 50% following administration of CSA. These changes were reflected in significant reductions in the number of liver granulomas found in drug-treated and control mice, except in the case of CSA, which showed no difference from control (Fig. 5B). The largest reduction in granuloma number per cm^2^ (45%) was found for dexverapamil. We also observed a significant reduction in granuloma size when infected mice were treated with any of the four Pgp inhibitors (Table 1). To determine whether the effects of these drugs on parasite egg production persist outside of the host, we perfused adult worms from C-4-, dexverapamil-, and CSA-treated mice and measured subsequent egg production during culture *ex vivo*. These cultured adult worms do not show a significant decrease in egg production, except for those parasites perfused from mice that had been treated with CSA (Fig. 5C).

**Table 1:** Mean granuloma area (µm^2^ ± s.e.m) in livers from *S. mansoni*-infected mice treated with Pgp inhibitors.

*Control*	*C-4*	*Dexverapamil*	*CSA*	*Tariquidar*
*(n = 31)*	*(n = 33)*	*(n = 24)*	*(n = 34)*	*(n = 29)*
3593 ± 381.4	2670 ± 287.8*	1562 ± 177.7^***^	2313 ± 287.8^**^	2525 ± 236.1*

*, **, and *** indicate P<0.05, P<0.01, and P<0.001, respectively, compared to Control, ANOVA. n  =  number of granulomas measured.

## Discussion

In this report, we used genetic and pharmacological approaches to disrupt normal MDR transporter function in *S. mansoni*. Strikingly, both approaches produced quite similar phenotypes. Knockdown in adult schistosomes of SMDR2, SmMRP1, or both resulted in a marked reduction in parasite egg production *ex vivo*, as did exposure of adult worms to the different MDR inhibitors. Notably, schistosomes residing within the murine host were also apparently susceptible to disruption of MDR function. *S. mansoni*-infected mice treated with any of four different Pgp inhibitors, including the potent third-generation inhibitor tariquidar, showed significant reductions in parasite egg burden in their livers. These results point to an essential role for ABC-type MDR transporters in schistosome reproduction.

Previous studies by us and others have investigated the involvement of these transporters in PZQ action and susceptibility. For example, we showed that PZQ interacts directly with the *S. mansoni* Pgp orthologue SMDR2, acting to both inhibit substrate transport, and as a likely substrate itself [43]. Furthermore, both SMDR2 and SmMRP1 are upregulated in response to PZQ and higher expression of these transporters is associated with reduced PZQ susceptibility [21], [42]. Here, however, we show that SMDR2 and SmMRP1 additionally appear to play important roles in schistosome reproductive physiology.

Though the MDR inhibitors we used in these experiments are structurally diverse and have wholly different molecular targets and modes of action, one characteristic they share is that they all inhibit mammalian Pgp or MRP1. CSA has previously been shown to have schistosomicidal activity at higher concentrations, most potently during the early course of infection [56]. This activity appears to be independent of the drug's immunosuppressive properties [56], [57], and the precise mode of the drug's antischistosomal action remains largely undefined [58]. CSA has also been shown to “sterilize” worms when administered every day over an eight-day period to *S. mansoni*-infected mice (days 28–35 post-infection), essentially eliminating liver egg burden [59], a result comparable to ours. CSA also enhances the pulmonary granuloma response in egg injection assays [60], which appears to be consistent with the lack of reduction we observe in the number of liver granulomas in CSA-treated infected mice (Fig. 5B). Interestingly, CSA was the only drug treatment in infected mice that appeared to have significant lasting effects on schistosome egg production after parasites had been removed from the CSA-treated host (Fig. 5C), perhaps indicating a long-lived or irreversible effect on reproductive physiology.

A second drug we used, dexverapamil, is an enantiomer of verapamil that is far less active against L-type Ca_v_ channels than the active enantiomer, but which retains potent inhibitory activity against mammalian Pgp. It too significantly disrupted egg production. Interestingly, a racemic mixture of verapamil was previously claimed in a patent to reduce egg production in *S. mansoni* [46], and we have confirmed that finding. The reduction in egg production following exposure of worms to dexverapamil, along with our results showing that verapamil is a potent inhibitor of SMDR2 [43], point to inhibition of *S. mansoni* Pgp, and not disruption of Ca_v_ channel function, as a likely mode of action.

C-4 is a derivative of curcumin that reverses the MDR phenotype and that reversibly inhibits mammalian Pgp transport of rhodamine [50]. Interestingly, curcumin, which also reverses MDR [61]–[63], has been shown to have antischistosomal activity at high (50–100 µM) concentrations and to reduce parasite egg production *ex vivo* at lower (5–10 µM) concentrations [64]. Tariquidar is one of the third-generation Pgp inhibitors developed specifically for high potency and selectivity against Pgp, and it completely eliminates *S. mansoni* egg production *ex vivo* at concentrations ≥12.5 µM. Finally, MK 571, an MRP1 inhibitor, also disrupts egg production.

Pairing of male and female worms is required for normal development and maturation of female schistosomes (reviewed in [65]). Thus, it is possible that the MDR inhibitors primarily affect male worms, and indirectly affect egg production in females. However, all of the Pgp inhibitors we tested decrease egg production in female worms cultured in the absence of males (Fig. 3F), and treated females are not “rescued” by addition of untreated males to the culture. Thus, inhibition of egg production does not appear to be due to effects of the drugs on male worms, and pairing is not required for those effects to appear.

All four of the Pgp inhibitors we tested *ex vivo* reduce liver egg burden in *S. mansoni*-infected mice. Use of other drug concentrations or routes of administration may enhance this effect and reduce pathology more dramatically. However, the fact that both genetic (RNAi) and pharmacological interference with normal MDR transporter function in schistosomes affects egg production suggests a common mode of action underlying this outcome, and that proper functioning of the parasite reproductive system may be dependent on MDR transporter activity. Furthermore, since excretion of eggs is essential for parasite transmission, and since host responses to egg deposition represent the major source of pathology in chronic schistosomiasis, disruption of egg production by interference with MDR transporter function could signal a vulnerability for exploitation in development of new antischistosomal therapeutics that exploit a multifaceted approach to reduce morbidity and the spread of the disease [66]. Furthermore, since higher levels of schistosome MDR transporters are associated with reduced PZQ susceptibility [21], [42], it will be interesting to determine whether knockdown or inhibition of these transporters potentiates the antischistosomal activity of PZQ.

## Materials and Methods

### Ethics statement

This study was carried out in strict accordance with the recommendations in the Guide for the Care and Use of Laboratory Animals of the U.S. National Institutes of Health. Animal handling and experimental procedures were undertaken in compliance with the University of Pennsylvania's Institutional Animal Care and Use Committee (IACUC) guidelines (Animal Welfare Assurance Number: A3079-01; IACUC protocol number 802105).

### Reagents

C-4 [(E)-4-Chloro-N-(3-(3-(4-hydroxy-3-methoxyphenyl)acryloyl)phenyl)benzamide] was from EMD Biosciences and cyclosporin A was from Enzo Life Sciences. R(+)-verapamil HCl (dexverapamil) and MK 571 were from Sigma-Aldrich. Tariquidar was from MedKoo Biosciences. The mouse monoclonal antibodies against Pgp (C219) and MRP1 (ab3371) were from Abcam. The anti-rabbit tubulin antibody was from Santa Cruz Biotechnology (H-235). Suppliers of molecular biology reagents are designated within the text.

### Isolation of schistosomes

Female Swiss Webster mice infected with *S. mansoni* (NMRI strain) obtained from the NIAID Schistosomiasis Resource Center at the Biomedical Research Institute in Rockville, MD were perfused 6–7 weeks post-infection, as described [67]. Perfused worms were maintained in RPMI (Invitrogen) plus 10% FBS (Sigma), 1% penicillin/streptomycin, and 0.012% Timentin (Plantmedia) at 37°C and 5% CO_2_.

### RNA interference

Knockdown of RNAs encoding SMDR2 (NCBI Acc. # L26287) or SmMRP1 (NCBI Acc. #GU967672) was as described [68], [69]. Briefly, following an overnight incubation in RPMI, adult worms (5 males plus 5 females) were placed in a 0.4 cm electroporation cuvette (USA Scientific Plastics) containing 50 µl siPORT (Ambion) and 3 µg SMDR2 siRNA (IDT), SmMRP1 siRNA (IDT), or luciferase siRNA (Ambion). For electroporation, a 20 ms square wave pulse of 125 volts was applied. The siRNAs were designed using IDT SciTools RNAi Design and the target sequences used in the studies were: SmMRP1 siRNA, 5′- GACCAATCAGCTAACCATAAATTTGTT- 3′, which maps to bp 3834–3860 of the SmMRP1 coding region RNA; and SMDR2 siRNA, 5′-TCGATCAAACCAACCAATCTCCTGTTT- 3′, which maps to bp 2332-2358 of the SMDR2 coding region RNA. The luciferase siRNA used for our control shows no significant similarity to any sequences from the *S. mansoni* gene database. Following electroporation, worms were incubated *en masse* in RPMI medium for 2 days. They were then sorted into 2–3 males/female pairs per well in a 12-well plate, in which they were maintained for an additional 48 to 72h, and subsequently removed from the medium, quick-frozen in liquid nitrogen, and stored at −80°C until further use. The number of eggs deposited in each well by these worms over this 48 to 72 h period was counted (see below).

### RNA and protein extractions

Total RNA was extracted as described [42], using either RNAqueous-4-PCR (Ambion) or NucleoSpin RNA XS (Macherey-Nagel), and subsequently treated with Turbo-DNAase (Ambion) or rDNAase (Macherey-Nagel) according to the manufacturer's instructions. For protein extractions, worms were homogenized in cell disruption buffer (Ambion Paris Kit) with a cocktail of protease inhibitors (Sigma) at 4°C and incubated for 15 min on ice. Lysates were centrifuged at 13,000 rpm for 10 min at 4°C and the supernatant collected was used immediately or stored at −20°C. Total protein concentrations were measured using a Bradford assay (Fermentas) with BSA (Sigma) as a standard.

### Real-time RT-PCR

Real-time RT-PCR was used to measure RNAi knockdown. It was performed using the Brilliant II SYBR green qRT-PCR Master kit (Stratagene) on an Applied Biosystems 3500 according to the manufacturer's recommendations. Primers used for the amplification of SMDR2, SmMRP1 and 18S ribosomal RNA have been described previously [21], [42]. Data were analyzed using the 2^−ΔΔ^C_t_ method [70] to determine the relative expression ratio between target (SmMRP1, SMDR2) and reference genes (18S RNA).

### Immunoblotting

Knockdown was also measured at the protein level by immunoblotting. Protein samples (25 µg) were electrophoresed on Bis-Tris gels in MOPS running buffer (Invitrogen), blotted, and probed with anti-Pgp, anti-MRP, or anti-β-tubulin antibodies, as described [21], [42].

### Treatment of worms with pharmacological compounds

Drugs were dissolved in dimethyl sulfoxide or ethanol for stock solutions, which were subsequently diluted 1:1 into Cremophor-EL (Sigma), and finally to an appropriate concentration in culture media. For *in vitro* treatments, 2 or 3 adult worm pairs were incubated in our standard media with different concentrations of drug (or carrier for controls) for two days. For treatments of *S. mansoni*-infected mice, drugs dissolved in 1:1 DMSO/Cremophore-EL (or carrier alone for controls) were diluted to 100 or 200 µl in PBS and administered intraperitoneally to mice beginning at 5–6 weeks post infection with approximately 200 cercariae. Each infected mouse was treated once per day on three alternate days with R(+)-verapamil (dexverapamil) HCl (60 mg/kg), C-4 (50 mg/kg), tariquidar (15 mg/kg) or cyclosporin A (60 mg/kg).

### Egg counts *ex vivo*

Worms subjected to different treatments were placed in individual wells of a multiwell plate, with 2–4 worm pairs (male + female) per well, and maintained in our standard worm culture medium at 37°C and 5% CO_2_. As reported by others [45], [71], adult worms perfused from mice will produce eggs while cultured *ex vivo*. At various times (typically 2d or 5d), we counted the cumulative number of eggs produced from treated and control worms, using a dissecting microscope. The number of eggs per control females typically ranged from 20 to 100, and varied within that range between different batches of perfused worms. For that reason, for all experiments except those in Fig. 3F, worm counts within each experiment were normalized to the mean value for the control worms for that experiment. The state of pairing of males and females was dynamic over the course of the incubation; paired worms would often separate, and these separated worms would often become paired again, In addition to obtaining egg counts, abnormal morphology of eggs was also noted and photographed. Adult schistosomes were fixed, stained with hydrochloric carmine (Sigma), and examined on a Leica SP5 two-photon confocal microscope, as described [72], [73].

### Enumeration of egg burden in mouse livers

Approximately 24 h following drug treatment, and while simultaneously collecting adult schistosomes, livers from drug-treated and control mice were isolated and weighed. A 0.25–0.5 g portion from the equivalent lobe of liver from different treatment conditions was dissected and incubated in 4% KOH for 16 to 24 h at 37°C as described [67]. The suspensions were examined for *S. mansoni* eggs, which were sampled and counted under a dissecting microscope multiple times for each mouse, and the number of eggs per gram of liver calculated. In some experiments, egg numbers were also corrected for the number of females perfused from each mouse, though that value did not vary significantly between the different treatments. In order to correct for variation between experiments, average egg counts per gram of liver within each experiment were normalized, with the mean control value set as 1. Remaining liver tissue was formalin-fixed, paraffin-embedded, and stained with haematoxylin and eosin. Granulomas within a set area of the sections were counted and the number of granulomas per cm^2^ calculated. To calculate granuloma area, the diameter of those granulomas surrounding a single egg from each section were measured using QCapture Pro software. Granulomas were assumed to be a spherical shape [74] and sizes calculated from the different fields of the histopathological sections.

### Statistics

Data are expressed as mean ± SEM, and were tested for statistical significance using either ANOVA or unpaired *t*-tests, as noted in the figure legends.

## Supporting Information

Figure S1 **Dexverapamil exposure alters the morphology of the female reproductive system.** Confocal micrograph of untreated (A) and 10 µM dexverapamil-treated (B) female *S. mansoni*. Following a 48 h incubation, worms were carmine-stained and examined by laser-scanning confocal microscopy as described [72], [73]. Arrows indicate cluster of developing, immature oocytes in control (A) vs. the second cluster of apparently mature oocytes in the dexverapamil-treated worms (B). Scale bar is 25 µm.
(TIF)
